# Quorum-Quenching Bacteria Isolated From Red Sea Sediments Reduce Biofilm Formation by *Pseudomonas aeruginosa*

**DOI:** 10.3389/fmicb.2018.01354

**Published:** 2018-07-17

**Authors:** Zahid Ur Rehman, TorOve Leiknes

**Affiliations:** Water Desalination and Reuse Center, King Abdullah University of Science and Technology, Thuwal, Saudi Arabia

**Keywords:** quorum quenching, marine bacteria, *N*-acylhomoserine lactone degradation, Red Sea sediments, biofilm inhibition

## Abstract

Quorum sensing (QS) is the process by which bacteria communicate with each other through small signaling molecules such as *N*-acylhomoserine lactones (AHLs). Certain bacteria can degrade AHL molecules by a process called quorum quenching (QQ); therefore, QQ can be used to control bacterial infections and biofilm formation. In this study, we aimed to identify new species of bacteria with QQ activity. Red Sea sediments were collected either from the close vicinity of seagrass or from areas with no vegetation. We isolated 72 bacterial strains, which were tested for their ability to degrade/inactivate AHL molecules. *Chromobacterium violaceum* CV026-based bioassay was used for the initial screening of isolates with QQ activity. QQ activity was further quantified using high-performance liquid chromatography-tandem mass spectrometry. We found that these isolates could degrade AHL molecules of different acyl chain lengths as well as modifications. 16S-rRNA sequencing of positive QQ isolates showed that they belonged to three different genera. Specifically, two isolates belonged to the genus *Erythrobacter*; four, *Labrenzia*; and one, *Bacterioplanes*. The genome of one representative isolate from each genus was sequenced, and potential QQ enzymes, namely, lactonases and acylases, were identified. The ability of these isolates to degrade the 3OXOC12-AHLs produced by *Pseudomonas aeruginosa* PAO1 and hence inhibit biofilm formation was investigated. Our results showed that the isolate VG12 (genus *Labrenzia*) is better than other isolates at controlling biofilm formation by PAO1 and degradation of different AHL molecules. Time-course experiments to study AHL degradation showed that VG1 (genus *Erythrobacter*) could degrade AHLs faster than other isolates. Thus, QQ bacteria or enzymes can be used in combination with an antibacterial to overcome antibiotic resistance.

## Introduction

Quorum sensing (QS) is the molecular mechanism by which bacteria monitor their population density in the local environment and regulate their behavior in a collective manner (Fuqua et al., 1994). QS is achieved by bacteria through the production of small chemical signaling molecules, collectively known as auto-inducers. Bacteria produce various kinds of auto-inducers that differ in chemical structure and mechanism of action. Broadly, auto-inducers are categorized into three types: (i) acylhomoserine lactones (AHLs), (ii) auto-inducing peptides (AIPs), and (iii) auto-inducer 2 (AI-2) (Huang et al., 2016). QS is used by bacteria to regulate biofilm formation, conjugal DNA transfer, pathogenesis, production of extracellular polysaccharides, and other processes (Galloway et al., 2011). QS blockade is hypothesized to be of use to control infections and biofilm formation by bacteria.

Quorum quenching (QQ) refers to the mechanism by which bacterial communication can be interrupted. QQ can be achieved by inhibiting the production of auto-inducers, their detection by receptors, or their degradation (Natrah et al., 2011). Interference of QS by blocking signal production is not very common and few reports discuss this approach (Hentzer and Givskov, 2003). Many organisms such as algae (Givskov et al., 1996), plant (Gao et al., 2003), and bacteria (Teasdale et al., 2009) produce molecules that are structurally similar to AHLs, and therefore, competitively inhibit their binding to receptors. Certain mammalian cells (Yang et al., 2005) and bacteria (Dong and Zhang, 2005; Romero et al., 2011; Torres et al., 2016) produce enzymes that can degrade or modify AHLs. Bacteria from both terrestrial and marine environments are known to produce AHL-degrading/modifying enzymes (Dong et al., 2002; Romero et al., 2008). The widespread prevalence of QQ enzymes in bacterial communities suggest that it provides competitive advantage to the producer in terms of food and space.

In the wake of rising antimicrobial resistance and toxic impact of antimicrobials on the environment, it is necessary to explore alternative methods to control bacterial infections. QQ is one such alternative, which has been successfully tested in diverse industries (Bzdrenga et al., 2017). For example, QQ has been successfully employed to reduce the pathogenicity of common plant pathogens (Zhang et al., 2007). Similarly, QQ can reduce membrane biofouling in wastewater treatment plants (Oh et al., 2012; Kim et al., 2015; Huang et al., 2016). Successful utilization of QQ in lab-scale wastewater treatment plants has allowed its application in large pilot-scale wastewater treatment plants (Lee et al., 2016). In aquaculture industry, QQ has shown positive results in the disruption of bacterial infections (Cao et al., 2012; Romero et al., 2014; Vinoj et al., 2014; Torres et al., 2016). Recently, QQ was tested for its ability to mitigate the biofouling of reverse osmosis membranes used in seawater desalination (Oh et al., 2017). QQ also has other potential applications such as control of biofouling on the hulls of shipping vessels and fishnets and bio-corrosion of oil production wells. Therefore, there is a need to identify new/novel bacterial species that can produce robust enzymes for use in non-conventional environments; our study is an attempt toward this.

Bacteria can produce three different types of enzymes that can degrade or modify AHLs (Dong and Zhang, 2005): AHL-lactonases hydrolyze the lactone moiety of AHLs (Dong et al., 2001), AHL-acylases hydrolyze the amide bond between lactone ring and acyl chain (Lin et al., 2003), and AHL-oxidoreductase oxidize or reduce the third carbon of the acyl chain of AHL molecules. Generally, hydrolysis of AHL molecules results in complete loss of activity, while oxidation/reduction reduces their activity (Chowdhary et al., 2007). This suggests that lactonases and acylases are more potent and useful in inhibiting bacterial communication.

Quorum quenching is gaining importance as a new way to control bacterial biofilms in medical and industrial domains, aquaculture, and water treatment plants (Torres et al., 2016; Bzdrenga et al., 2017). In this study, we attempted to isolate bacteria from sea sediments that can degrade AHLs and interfere with bacterial communication. We focused on QQ based on AHL inhibition because AHL-based QS is predominantly used by gram-negative bacteria, which are the dominant bacteria found in marine environments and are regarded as early colonizers during biofilm formation (Dang and Lovell, 2000; Zhang et al., 2006). For bacterial isolation, we used sediments from the Red Sea because this particular niche has not been explored from the point of view of QQ. Furthermore, this niche might help us identify new/novel species of bacteria that can be used for biofilm control for applications wherein terrestrial bacteria cannot be used. Screening of these isolates helped identify bacteria with QQ activity. Sequencing the genomes of these isolates allowed us to identify open reading frames (ORFs) encoding QQ enzymes. We further showed that these isolates can be used to degrade a wide range of AHL molecules as well as inhibit biofilm formation by *Pseudomonas aeruginosa* PAO1.

## Materials and Methods

### Sample Collection and Isolation of Bacteria

Red Sea sediment cores were collected at a depth of 1–2 m from the coastal area (22.389778 and 39.135556) 12 km north of Thuwal, Saudi Arabia, in February 2016. Samples were collected from two different areas: one with vegetation (seagrass) and one without vegetation. Sediments were sampled using 30-cm-long acrylic cylindrical tube with a diameter of 5 cm. An ∼20-cm sediment core was collected, and the remaining headspace was filled with indigenous seawater. After sediment collection, rubber stoppers were inserted to seal the two ends of the cylinder. Sampled sediments were stored at 30°C and used to isolate QQ bacteria at the earliest to avoid any negative effect of storage. About 1 g of sea sediments collected from a depth of 2 cm from the surface of the sampling cylinder was suspended in 1 mL of 0.2-μm filtered autoclaved seawater and vortexed. Samples were allowed to stand for 1–2 min to allow the particles to settle down. The supernatant was then subjected to 10-fold serial dilution. Each dilution was plated on Marine Agar (MA) (HIMEDIA, India), R2A agar (HIMEDIA, India), and Casamino acids (CAS) agar (VWR, United States). Both R2A and CAS agar were prepared in 75% of 0.2-μm-membrane-filtered autoclaved seawater. The plates were incubated at 30°C for 1 week. Colony-forming units observed on plates (with 30–300 colonies) were enumerated, and the colonies were further subcultured onto sterile agar plates based on macroscopic characteristics. Single colonies were further streaked twice to obtain pure cultures.

### QQ Assay

The isolated strains were tested for QQ activity by using the AHL biosensor strain *Chromobacterium violaceum* CV026. This sensor strain has been used to detect C6-AHLs in various studies (McClean et al., 1997; Romero et al., 2011; Torres et al., 2016). The isolates were grown in 0.5 mL of the isolation medium and incubated at 30°C with shaking at 150 rpm. C6-AHLs were added to this bacterial culture to reach a final concentration of 10 μM (2 μg/mL) and further incubated for 24 h at 30°C with shaking. The pH of this mixture was measured to confirm that the observed degradation of AHLs was not caused by alkaline pH (Yates et al., 2002). C6-AHLs mixed with cell-free medium were used as the negative control. The bacterial cultures were centrifuged to pellet the cells, and the remaining C6-AHLs in the culture supernatant were detected by the following method. Luria-Bertani (LB) agar plates were overlaid with 5 mL of 1/100th-dilution of an overnight culture of the biosensor strain CV026 mixed with LB soft agar (0.7%). After the biosensor layer was solidified, 6-mm wells were created in the medium by using sterile pipette tips. These wells were filled with the culture supernatant and incubated at 30°C for 24 h. Solvent without C6-AHLs was used as the blank. The appearance of a purple halo around the well-indicated the absence of QQ activity. On the other hand, strains with QQ activity degraded C6-AHLs, and therefore, the biosensor strain was not activated. Thus, halo formation was not observed. Furthermore, the culture supernatant of QQ isolates was tested for the production of C6-AHLs.

No purple halos were observed in the CAS and R2A cell-free media (negative controls), which showed that these media cannot be used for QQ assay. Therefore, for these isolates, we slightly modified the QQ assay, as described previously (Uroz et al., 2005; Shepherd and Lindow, 2009). Briefly, 24-h-old bacterial cultures were centrifuged to obtain cell pellets. These pellets were suspended in 0.5 mL of 1X phosphate-buffered saline (PBS) containing 10 μM C6-AHLs and incubated overnight at 30°C with shaking. The remaining procedure was as described above.

### QQ Assay With Heat-Inactivated Bacteria

To ensure that the loss of C6-AHL activity observed in QQ-positive strains was not due to the adsorption of these molecules onto the cell surface, the bacterial cells were heat killed. Bacterial cells were heated at 100°C for 15 min. Heat-killed bacterial cells were allowed to cool down for 10 min at room temperature. QQ assay was performed as described above. Bacterial cell death was confirmed by plating 150 μL of the heat-treated cell suspension on respective culture medium.

### Detection and Localization of AHL-Degradation Activity

This assay was performed as described previously with slight modifications (Romero et al., 2008; Torres et al., 2016). About 200 mL of the overnight culture suspension of QQ-positive isolates was centrifuged at 7000 ×*g* for 10 min. Cell pellets were washed with an equal volume of 1X PBS and re-suspended in 50 mL of PBS. Cells were lysed by intermittent ultra-sonication (Qsonica, United States) for 5 min in a cold water bath at a frequency of 15 kHz. Lysed cells were centrifuged at 16000 ×*g* for 30 min at 4°C. Cell lysates were filtered through a 0.2-μm-pore-sized-membrane filter. The protein concentration of the cell lysates was determined with Qubit (Invitrogen, United States). To determine AHL-degradation activity, 500 μL of the cell lysates was incubated with 10 μM C6-AHLs for 24 h at 30°C, with shaking at 140 rpm. The remaining C6-AHLs were detected by a well-diffusion agar plate assay, as described above. Cell lysate without C6-AHLs was used as the control. To understand the chemical nature of QQ activity, the cell lysates were heated at 95 and 105°C for 10 min. Furthermore, the cell lysates were fractionated using 10-kDa centrifugal filters (Amicon, United States) and QQ activity was analyzed for both the retentate and filtrate of cell lysates.

### HPLC-MS-Based Analysis of AHLs

The ability of isolates to degrade different types of AHLs was studied by using high-performance liquid chromatography-tandem mass spectrometry (HPLC-MS) as described previously (Romero et al., 2011). Briefly, overnight bacterial cultures were centrifuged and re-suspended in PBS containing 10 μM AHL. This mixture was incubated overnight at 30°C with shaking. For the time-course experiment, the samples were withdrawn every hour for 5 h. To extract AHLs, the cells were separated by centrifugation at 7000 ×*g* for 5 min, and the PBS was extracted twice with an equal volume of ethyl acetate (Fisher Scientific, United States). Ethyl acetate was evaporated under a flux of nitrogen at 40°C, and the final extract was suspended in 400 μL of acetonitrile (Fisher Scientific, United States) for HPLC-MS. PBS containing equal amount of AHLs was used as the negative control. To determine whether the QQ activity was caused by the hydrolysis of lactone ring (lactonolysis), the bacterial cells were incubated overnight with PBS containing 50 μM 3OHC10-AHLs. The cell-free supernatant was acidified to a pH of 2 by adding 10 mM hydrochloric acid (HCl). The acidified supernatant was incubated overnight at room temperature to allow re-cyclization of lactone ring. AHLs were extracted from this solution as described above.

High-performance liquid chromatography 1100 series equipped with ZORBAX Eclipse XDB-C18 (4.6 mm × 250 mm column; 5-μm particle size; Agilent Technologies, United States) kept at 45°C was used for analysis. About 10 μL of the extract was injected at a flow rate of 0.45 mL/min. For elution, a mobile phase consisting of solvent B (methanol with 0.1% formic acid) and solvent A (25 mM ammonium formate with 0.1% formic acid) was used. The gradient profile used was 1 min of 10% solvent B, followed by a linear gradient gradually increasing to 95% of solvent B over 15 min. Solvent B (95%) was then stabilized for 4 min. The column was re-equilibrated for a total of 5 min. MS data were obtained on TSQ Vantage triple-quadruple mass spectrometer (Thermo Fisher Scientific, United States) by using positive-ion electrospray and multiple-reaction-monitoring (MRM) mode.

### Bacterial Identification Based on 16S-rRNA Gene Sequencing

About 500 μL of the overnight bacterial suspension was centrifuged and the cell pellets were re-suspended in 500 μL of nuclease-free water. Bacterial cells were lysed by heating at 95°C for 10 min, followed by cooling for 15 min at room temperature. The lysed bacterial cells were centrifuged at 12000 ×*g* for 3 min, and 1 μL of the supernatant was used as template DNA for polymerase chain reaction (PCR). A set of three primer pairs, namely, (27F-785R), (341F-907R), and (785F-1492R) was used to amplify the I6S-rRNA gene. Primer sequences are available in Supplementary Table 4. Following PCR conditions were used: initial denaturation at 95°C, followed by 30 cycles of denaturation at 94°C for 30 s; primer annealing at 52°C (27F-785R), 62°C (341F-907R), and 53°C (785F-1492R) for 30 s; and extension at 72°C for 1 min. Final extension was performed at 72°C for 5 min. The PCR product was analyzed by gel electrophoresis and purified using the ExoSap-IT PCR product cleanup kit (Affymetrix, United States), according to manufacturer’s instructions. The purified DNA was submitted for Sanger sequencing. The three overlapping sequences were aligned to obtain a single rRNA molecule for use in BLAST search (Altschul et al., 1997) against the 16S-rRNA gene sequences available in the GenBank database. The 16S-rRNA gene sequences of close relatives, as determined by BLAST and the QQ bacteria described in literature, were used for phylogenetic analysis.

For phylogenetic analysis, the SINA software package available in SILVA rRNA database (Quast et al., 2013) was used to align 16S-rRNA gene sequences. The aligned sequences were subjected to phylogenetic tree construction by using MEGA7 (Kumar et al., 2016) software at default parameters.

### Acylhomoserine Lactones (AHLs)

Following AHLs were used in this study; *N*-butyryl-DL-homoserine lactone (C4-AHLs), *N*-hexanoyl-DL-homoserine lactone (C6-AHLs), *N*-decanoyl-DL-homoserine lactone (C10-AHLs), *N*-tetradecanoyl-DL-homoserine lactone (C14-AHLs), *N*-(3-oxodecanoyl)-DL-homoserine lactone (3OXOC10-AHLs), *N*-(3-hydroxydecanoyl)-DL-homoserine lactone (3OHC10-AHLs), and *N*-(3-oxododecanoyl)-L-homoserine lactone (3OXOC12-AHLs). All AHLs used in this study were purchased from Sigma, United States.

### Biofilm Formation and Quantification

The impact of QQ bacteria on biofilm formation by *P. aeruginosa* PAO1 was studied using a recently described segregated culture bioassay (Oh et al., 2017). In this assay, QQ bacteria are physically separated from PAO1 by using a semipermeable membrane (Transwell polycarbonate membrane cell inserts; Corning, NY, United States). PAO1 (OD_600_ = 0.01) was directly inoculated into the wells of a 24- or 6-well microtiter plates. QQ bacteria (live or dead) were added into the membrane inserts and installed into the wells. As a control, QQ bacteria were killed by incubating the cells in 4% paraformaldehyde for 30 min at room temperature. Cell death was confirmed by spreading the cell suspension on R2A or MA plates. The inoculated microtiter plates were incubated for 24 h at 30°C with shaking at 60 rpm. The membrane inserts were removed, and the OD_600_ of the PAO1 cell suspension was measured to determine the effects of QQ bacteria on growth, if any. Biofilm formation by PAO1 on the wells was measured using the crystal violet assay (Coffey and Anderson, 2014). PAO1 culture was also used for the extraction and quantification of 3OXOC12-AHLs, as described above. Furthermore, 3OXOC12-AHLs were quantified in the control sample (LB) as well as VG1, VG12, and NV9.

### Genome Sequencing and Annotation

Genomic DNA of the strains to be sequenced was extracted using QIAGEN genomic-tip 100/G columns (QIAGEN, Germany). A genome-sequencing library was prepared using the Pacific Biosciences (PacBio) 20-kb template preparation kit by employing the BluePippin size selection system and sequenced on PacBio RS platform. The PacBio reads were assembled using the CANU WGS assembler version 1.4 (Koren et al., 2017). The assembled genome was annotated using the Automatic Annotation of Microbial Genomes (AAMG) pipeline (Alam et al., 2013). For functional annotation, the predicted ORFs were compared to the latest version of UniProt (The Uniprot Consortium, 2017) and Kyoto Encyclopedia of Genes and Genomes (KEGG) (Kanehisa et al., 2014).

### Statistical Analysis

Mean and standard deviation were calculated for AHL degradation and biofilm inhibition assays. Further statistical analyses such as analysis of variance (ANOVA) with Bonferroni corrected *post hoc t*-test and also Student’s *t*-test were preformed where statistical significance was (*p*-value < 0.0063) or (*p*-value < 0.05) respectively. All the statistical analyses were performed in Microsoft^®^ Excel version 16.9.

## Results

### Bacterial Isolates

Different number of CFUs were obtained, from both types of sea sediment samples by using three different culture media. The CFU/gram of sea sediment is listed in Supplementary Figure 1. For all culture media, the number of CFUs obtained from the samples from non-vegetative areas was higher compared to that from the samples collected from the vicinity of vegetation (Supplementary Figure 1).

Higher CFUs were observed on MA medium, compared to R2A and CAS media. The highest number of CFUs, that is, 6.6 × 10^4^, was obtained from the samples obtained from non-vegetative areas that were plated on MA. For the samples collected from the vicinity of vegetation, 4.4 × 10^4^ CFUs were obtained on MA (Supplementary Figure 1), which was four-times higher than that obtained on R2A and CAS media. Isolates exhibiting different colony morphology were selected for QQ assay.

### Biosensor-Based Detection of QQ Activity

About 71 bacterial isolates were screened for QQ activity. A solid plate assay was performed using the biosensor strain *C. violaceum* CV026, which produces a purple pigment violacein in response to C6-AHLs (McClean et al., 1997). The QQ strains can degrade AHLs, which, in turn, did not allow the development of any color. The number of isolates tested and those testing positive for QQ activity is listed in **Table 1**. Of the 14 QQ-positive isolates, 64.3% were isolated on the R2A medium, followed by MA (21.5%) and CAS (14.3%). However, the QQ-positive CAS and MA isolates showed only partial degradation of C6-AHLs, as indicated by the small/faint purple halos (Supplementary Figures 2, 3). Most QQ-positive isolates obtained on R2A showed complete degradation of AHLs. Overall, 22.4% isolates from the samples obtained from areas near vegetation and 13.6% isolates from the samples obtained from areas without vegetation were positive for QQ activity (**Table 1**). C6-AHL production by the QQ isolates was not detected.

**Table 1:** Number and QQ activity of the strains isolated from different samples (vegetative and non-vegetative) by using different media.

Sample/medium	Isolates tested for QQ	QQ based on CV026
Vegetative		
MA	21	2
R2A	18	7
CAS	10	2
Non-vegetative		
MA	6	1
R2A	10	2
CAS	6	0
Total	71	14


*Number of isolates with positive QQ activity (based on C6-AHL degradation in the *Chromobacterium violaceum* CV026 assay) is shown.*

Some previous studies investigating the QQ potential of marine bacteria used marine broth for QQ assay (Romero et al., 2011; Torres et al., 2016). Therefore, we tried to cultivate the isolates obtained on R2A and CAS media in marine broth. However, except VG12, none of them grew in marine broth, although they did grow on MA. VG12 cultivated in marine broth continued to remain positive for QQ activity.

Quorum quenching assay performed using heat-killed QQ isolates ruled out the possibility that the observed loss of AHLs was due to adsorption onto bacterial cells (data not shown). Bacterial isolates that retained QQ activity after heat treatment were not included in further analyses. Of the 14 isolates, eight were selected for further analyses.

### QQ Analysis Based on HPLC–MS

**FIGURE 1:**
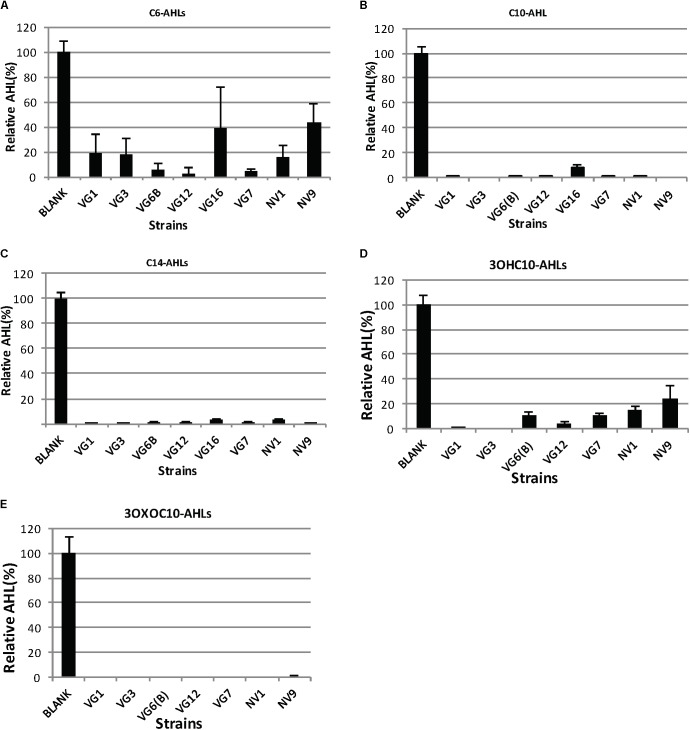
Degradation of AHLs by the isolated bacteria. The amount of AHLs degraded by different isolates is listed, relative to the negative control. Quantification of AHLs was performed as described in Section “Materials and Methods.” Briefly, AHLs were extracted with ethyl acetate, which was evaporated under a flux of nitrogen gas. The extracted AHLs were re-suspended in acetonitrile and quantified by HPLC-MS. Cell-free PBS was used as the negative control (100%). Values are the mean of three replicates; error bars represent standard deviation. Charts **(A–E)** illustrates the degradation of C6, C10, C14, 3OXOC10-AHLs, and 3OHC10-AHLs, respectively.

**FIGURE 2:**
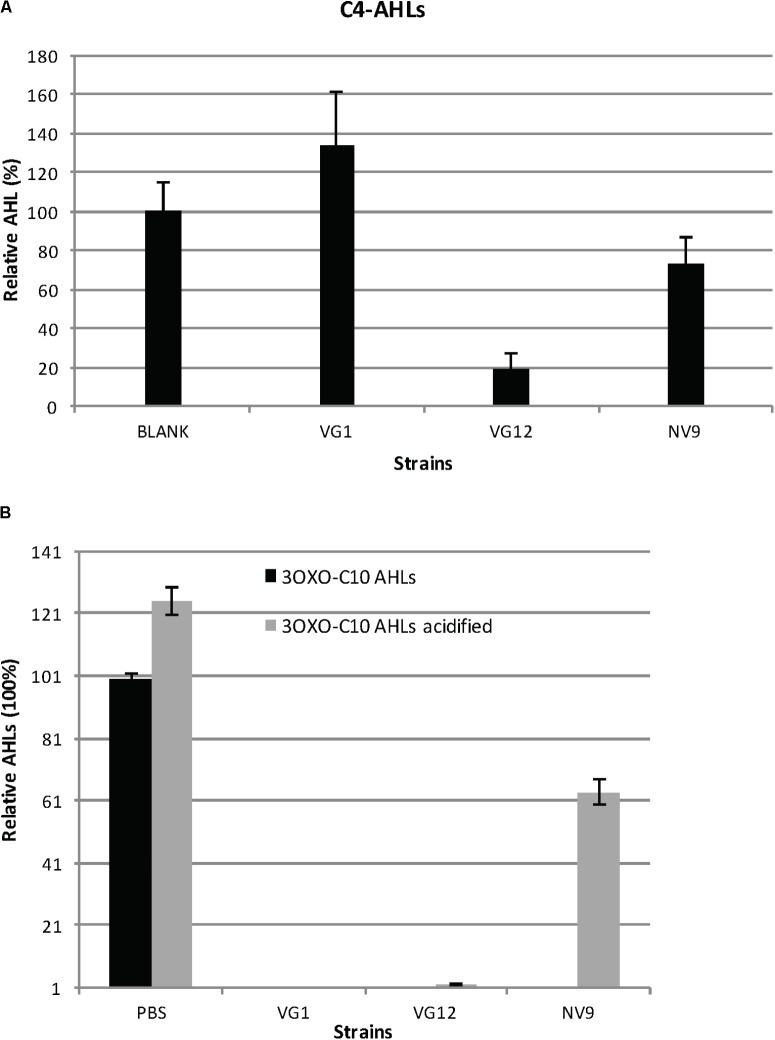
Degradation and acidification of AHLs. **(A)** Relative amount of C4-AHLs degraded by the three isolates is given. For quantification, the C4-AHLs were extracted with ethyl acetate and subsequently dried and re-suspended in acetonitrile for injection in HPLC-MS. Cell-free PBS served as the negative control (100%). Experiments were performed in triplicate; error bars represent the standard deviation of the mean value. Student’s *t*-test showed significant reduction in the amount of C4-AHLs by VG12 (*p*-value = 0.003) and NV9 (*p*-value = 0.03). No significant degradation of C4-AHLs by VG1 was observed (*p*-value = 0.11). **(B)** Acidification of 3OXOC10-AHLs after incubation with QQ bacteria. Relative amount of AHLs before and after acidification is given. Black bars represent the amount of AHLs after incubation with PBS (negative control is 100%) or QQ bacteria. Gray bars represent the amount of AHLs recovered after acidification. Error bars represent the standard deviation for the three independent replicates.

The QQ activity of the positive strains, as determined by the biosensor assay, was further confirmed by HPLC-MS. The ability of QQ bacteria to degrade different types of AHLs was also investigated. For this, QQ-positive bacterial cultures were mixed with AHLs of different acyl chain lengths and modifications (C4-AHLs, C6-AHLs, C10-AHLs, 3OXOC10-AHLs, 3OHC10-AHLs, and C14-AHLs). After 24 h of incubation, the final pH was < 7.5, which excluded the possibility of the hydrolysis of the lactone ring of AHL molecules due to alkalinity. The remaining AHLs were extracted and quantified (**Figures 1A–E, 2A**). All the strains showed significant reduction in the amount of AHLs, compared to the negative control (**Figure 1**). Analysis of variance (ANOVA) along with Bonferroni’s corrected *post hoc t*-test was applied, which showed significant reduction of AHLs by the QQ strains (*p*-value < 0.0063), compared to the blank sample. The degradation capacity of all isolates was higher for C10-AHLs and C14-AHLs, compared to C6-AHLs (**Figures 1A–C**). All QQ bacteria caused > 90% reduction in the quantities of C10 and C14-AHLs (**Figures 1A,B**). These results are in agreement with previous reports wherein the reduction in the amount of long-acyl-chain AHLs was higher compared to that in case of short-acyl-chain AHLs (Romero et al., 2008; Romero et al., 2011; Torres et al., 2016).

The ability of these QQ bacteria to degrade differently modified C10-AHLs (3OXO-AHLs and 3OH-AHLs) was also investigated (**Figures 1D,E**). The strain VG16 displayed inconsistent cultivability; therefore, it was not included in further analyses. In a recent study, most QQ bacteria were able to degrade a wide range of AHLs, but they could not effectively degrade 3OHC10-AHLs (Torres et al., 2016). Similar to this, all QQ-positive isolates in this study could degrade 3OXOC10-AHLs more effectively, compared to 3OHC10-AHLs (**Figures 1D,E**).

We also studied the ability of bacteria to degrade C4-AHLs. For this analysis, three QQ-positive bacteria (VG1, VG12, and NV9) belonging to different genera were selected. Of these, VG12 showed maximum degradation (>80 ± 8.9%) of C4-AHLs (**Figure 2A**), while NV9 showed only 26 ± 13% reduction and VG1 did not show significant degradation (**Figure 2A**).

To identify the nature of QQ activity, i.e., lactonase or acylase, 3OXOC10-AHLs degraded by VG1, VG12, and NV9 were treated with HCl. Acidification resulted in the reformation of lactone ring that suggested lactonase activity (Yates et al., 2002; Romero et al., 2008). In NV9, ∼63.5 ± 4% of 3OXOC10-AHLs was recovered after HCl treatment. In VG12 and VG1, only 2 ± 0.003 and 0.004 ± 0.009% of AHLs, respectively, were recovered after acidification (**Figure 2B**).

### QQ Activity and Its Localization

The location of QQ activity (extracellular or intracellular) was studied for VG1, VG12, and NV9. Cell-free supernatants and lysates were incubated with C6-AHLs. Cell lysates and culture supernatant of VG1 were able to degrade C6-AHLs (Supplementary Figure 2B). No QQ activity was detected in the culture supernatant and cell lysates of VG12 and NV9 (Supplementary Figure 2B). Heat treatment of the cell lysates of VG1 at 95 and 105°C did not result in loss of QQ activity. After fractionation of the cell lysates of VG1 by using 10-kDa filters, QQ activity was detected only in the retentate but not in the filtrate (data not shown). This suggested that the molecules responsible for QQ activity are larger than 10-kDa.

### Time-Course Experiment of AHL Degradation

**FIGURE 3:**
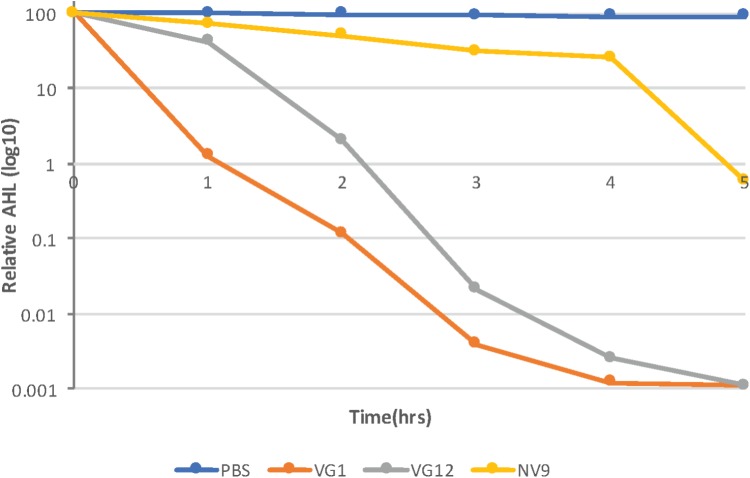
Time-course experiment to study AHL degradation. Log_10_ of the relative amount of AHLs quantified at each time point is given along the y-axis. AHLs were quantified using HPLC–MS, as described in Section “Materials and Methods.” The amount of AHLs at 0 h is considered 100%. Control (PBS) sample and different strains are represented by different colors, as indicated in the legend. Standard deviation at each time point was <10%.

The kinetics of the degradation of 3OXOC10-AHLs by VG1, VG12, and NV9 was also investigated. The isolate VG1 caused 98.7 ± 0.11% reduction in the first hour, while VG12 caused 58 ± 1.4% reduction and NV9 caused only 26.9 ± 8.2% reduction in the amount of AHLs (**Figure 3**). However, after 2 h, the amount of AHLs degraded by VG1 and VG12 was almost equal, i.e., 99.9 ± 0.01 and 98 ± 0.7%, respectively, while only 50 ± 3.4% of the AHLs was degraded by NV9. Maximum reduction of 3OXOC10-AHLs by NV9 occurred after 4 h (**Figure 3**).

### Identification of QQ Isolates

Phylogenetic analyses showed that all the seven QQ isolates belonged to the phylum Proteobacteria (Supplementary Table 1). Except NV9, all other isolates [VG1, VG3, VG6(B), VG12, VG7, and NV1] belonged to the class Alphaproteobacteria and two different genera *Erythrobacter* and *Labrenzia*. Isolate NV9 belonged to the class Gammaproteobacteria and genus *Bacterioplanes* (Supplementary Figure 4 and **Table 1**).

Isolates VG1 displayed 100% identity to *Erythrobacter flavus* SW-52, which was also isolated from the marine environment (Yoon et al., 2003). As described for *E. flavus*, VG1 formed yellow colonies on agar plates. Isolate VG3 showed 99% identity to *Erythrobacter* sp. JL-378 and also formed yellow colonies on R2A agar.

Four isolates, namely, VG6(B), VG12, VG7, and NV1, belonged to the genus *Labrenzia*. Different species of *Labrenzia* that were identified based on 16S-rRNA gene sequence homology are listed in Supplementary Table 1. VG12 showed 99% identity to *Alphaproteobacterium* JL001 that was isolated from marine sponges. Phylogenetic analysis showed that VG12 is closely related to the other *Labrenzia* species identified in this study and previously (Supplementary Figure 4). Isolate VG7 showed 99% identity to *Labrenzia* sp. A-3-20, which was recently isolated from the soft corals found in Baltic sea (Pham et al., 2016). NV1 displayed 99% identity to *Labrenzia* sp. R-666638. All species of genus *Labrenzia* that have been identified so far, have been isolated from marine environments (Biebl et al., 2007; Camacho et al., 2016).

The 16S-rRNA sequence of the QQ isolate NV9 (obtained from areas without vegetation) showed 99% identity to that of a recently proposed bacterial species *Bacterioplanes sanyensis* (Wang et al., 2014), also isolated from marine environment.

The phylogenetic relationship of the QQ isolates discussed in this study and other marine bacteria is illustrated in Supplementary Figure 4.

### Effect of QQ Bacteria on Biofilm Formation

**FIGURE 4:**
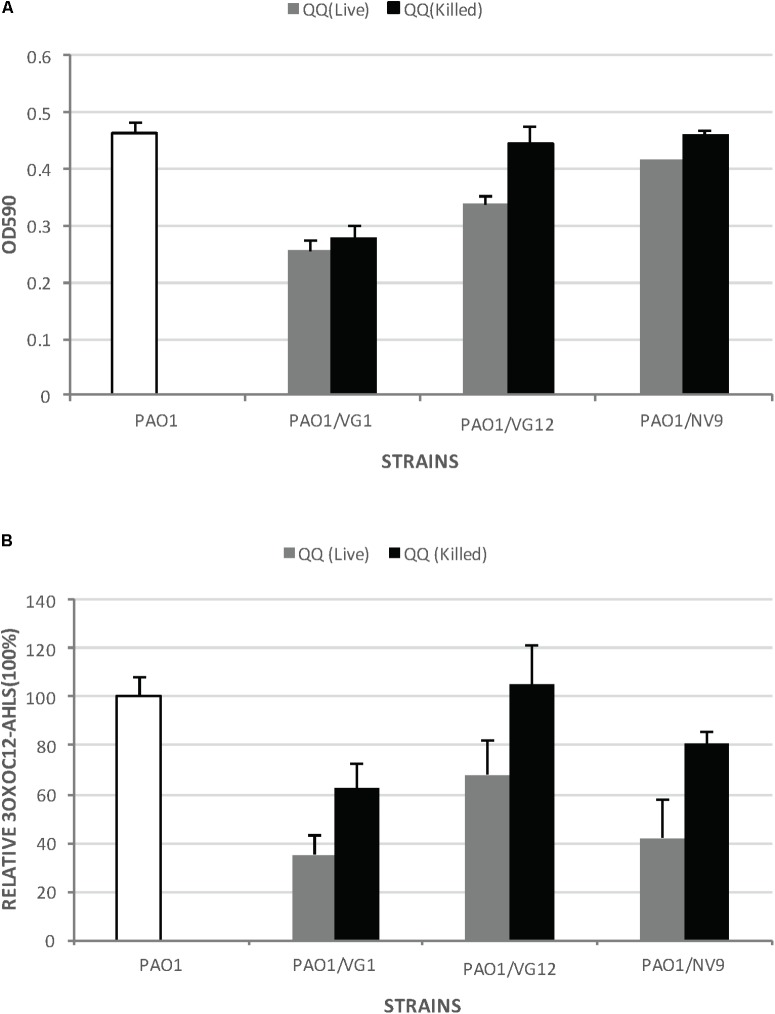
Biofilm formation by *Pseudomonas aeruginosa* PAO1 incubated with live and dead QQ strains. **(A)** This experiment was performed in microtiter plates with membrane inserts for wells, as described in Section “Materials and Methods.” The y-axis indicates the OD_590_ of the crystal violet bound to the wells. White bars represent biofilm formation by PAO1 without any live or dead QQ bacteria. Gray bars represent biofilm formation by PAO1 incubated with live QQ cells, while black bars represent biofilm formation by PAO1 incubated with dead QQ bacteria. LB broth was used as the negative control. Error bars represent the standard deviation for the three replicates. Student’s *t*-test was applied to determine significance; *p*-values: VG1 (0.29), VG12 (0.04), and NV9 (0.09). **(B)** Relative amount of 3OXOC12-AHLs in the supernatant of PAO1 incubated with live or dead QQ bacteria. The amount of AHLs in the supernatant of PAO1 incubated with live QQ bacteria is shown as gray bars, while that detected in the presence of dead QQ bacteria is shown as black bars. The amount of AHLs produced by PAO1 (without live/dead QQ bacteria) is shown by white bars (100%). Error bars represent the standard deviation. Student’s *t*-test showed no significant difference in the amount of 3OXOC12-AHLs in the PAO1 supernatant incubated with live/dead VG1 (*p*-value = 0.16), VG12 (*p*-value = 0.219), and NV9 (*p*-value = 0.22).

VG12 was able to significantly reduce biofilm formation by PAO1. Live VG12 cells could reduce biofilm formation by 25 ± 0.018% compared to dead VG12 cells (**Figure 4A**). However, no significant reduction was induced by VG1 and NV9 in the biofilm formation of PAO1.

*Pseudomonas aeruginosa* PAO1 produces 3OXO-C12AHLs, which directly or directly control the expression of virulence factors and biofilm formation (Williams and Camara, 2009). Therefore, the amount of 3OXO-C12AHLs in the supernatant of PAO1 incubated with live/dead QQ bacteria was also quantified. However, no significant degradation of 3OXO-C12AHLs was detected (**Figure 4B**). No 3OXO-C12AHLs were detected in case of LB, VG1, VG12, and NV9.

### Identification of Lactonases and Acylases in the Genome Sequences

For each strain, the genomic features and their counts are listed in Supplementary Table 3. The genome sequences were submitted to GenBank; the accession numbers for VG1 is CP022528, VG12 is CP022529, and NV9 is CP022530. Annotations for VG1 are available^1^, VG12^2^, and NV9^3^. Based on average nucleotide identity (ANI), a new quality control test implemented by GenBank, VG12 was designated as *Labrenzia* sp. VG12 because of its low similarity with the type strain *Labrenzia alba*.

Annotated genomes were searched for the homologs of AHL lactonases and acylases, which are members of the metallo-beta-lactamase (MBL) and N-terminal nucleophile hydrolases (Ntn-hydrolases) superfamilies, respectively (Utari et al., 2017).

VG1 genomic annotations showed that the two ORFs (VG1_000001122 and VG1_000002328) are KEGG orthologs of AHL-lactonases (K13075). UniProt annotations further confirmed that these proteins are beta-lactamases. Similarly, both KEGG (K07116) and UniProt annotations suggest that ORF VG1_000002924 is an AHL-acylase (**Table 2**).

**Table 2:** Genomic IDs of the ORFs of the sequenced strains, showing homology to AHL-lactonases or AHL-acylases.

Strains	ORFs (ID)	
	
	Lactonases	Acylases
VG1	VG1_000001122	VG1_000002924
	VG1_000002328	
VG12	VG12_000006578	
	VG12_000000021	
	VG12_000000913	
	VG12_000003727	
	VG12_000004165	
NV9		NV9_000000564


*Open reading frames predicted by both KEGG and UniProt are listed; the amino acid sequences of these ORFs can be accessed using the annotation links given in Section “Results.”*

For VG12, both KEGG Orthology and UniProt predicted that VG12_000000021, VG12_000006578, VG12_000000913, and VG12_000004165 belong to the lactonase group and/or are MBL members. The protein product (VG12_000003727) was predicted as AHL-lactonase by KEGG, but UniProt showed it to be Ribonuclease Z. BLAST analysis of this ORF showed that it is 90% identical to the MBL superfamily of proteins (**Table 2**). No homolog of AHL-acylases was identified for VG12, neither by KEGG nor UniProt.

For NV9, both KEGG and UniProt annotations indicated that NV9_000000564 is an AHL-acylase (**Table 2**).

Apart from these ORFs, the genomes of VG1, VG12, and NV9 carry other proteins that are homologous to MBL and amidases. The locus IDs of these ORFs are given in Supplementary Table 2.

## Discussion

The emergence of antimicrobial resistance has underscored the need to develop new strategies to control bacterial infections and biofilms. Furthermore, the environmentally toxic biocides used in water treatment, agriculture, and oil and shipping industry warrant the search of sustainable and non-toxic alternatives. QS is a potential target for use as a new therapeutic approach because of its role in bacterial infection and biofilm formation. One such opportunity can be identified by exploring QQ because of its potential benefits.

In this study, cultivable bacteria were isolated from Red Sea sediments collected from two different niches, i.e., areas with and without vegetation. Unexpectedly, a higher number of bacteria was isolated from the samples collected from areas without vegetation, which can be attributed to the fact that vegetative bacteria require the compounds produced by plants for their growth (Supplementary Figure 1). This can also be attributed to the inherent bias observed in the plate count method. By screening all isolates, we identified that ∼20% of the isolates exhibit QQ activity (**Table 1**). These results are similar to those of previous studies, which reported higher prevalence of QQ-positive bacteria in the marine environment, compared to the terrestrial environment (Romero et al., 2011; Saurav et al., 2016). It is important to note that we only used *C. violaceum* CV026-based assay for the initial screening of QQ bacteria, and thus, the number of positive isolates might be underestimated.

Quorum quenching bacteria have been detected and isolated from dense microbial communities in various systems (Tan et al., 2015; Saurav et al., 2016). Similarly, in this study, a higher percentage of QQ bacteria was detected from samples collected from areas with vegetation compared to those from areas without vegetation (**Table 1**). However, the vegetative bacteria identified in this study might not be permanently associated with seagrass because their close relatives have been isolated from different marine niches. It can also be that the microbial community associated with seagrass is dynamic, and that the QS and QQ activities play a role in the assembly of functional communities, as reported in case of tobacco rhizosphere and granular sludge community (d’Angelo-Picard et al., 2005; Tan et al., 2015). However, the biotechnological significance of QQ bacteria renders the association of these isolates with seagrass less important.

Not all QQ-positive isolates completely degraded the C6-AHLs involved in CV026 bioassay (Supplementary Figures 2A, 3). Moreover, some isolates did not show reproducible QQ activity, and thus, this inconsistency (Saurav et al., 2016) warrants further exploration of the regulatory mechanisms of expression of QQ activity. Isolates that displayed QQ activity even after heat killing (data not shown) indicate that either QQ activity is non-enzymatic and/or the loss of AHLs was due to adsorption onto cellular debris. However, it could also be attributed to the fact that the QQ is enzymatic and that these enzymes are heat resistant. A recent study has shown that Aii20J, an AHL-lactonase from *Tenacibaculum* sp. 20J, can retain its activity even after heating up to 100°C for 10 min (Mayer et al., 2015). We will investigate the possibility of heat-resistant enzymes in our future studies.

Based on our findings (**Figures 1, 2A**) and those of others (Romero et al., 2011; Torres et al., 2013; Tan et al., 2015), there appears to be a general feature: QQ bacteria capable of degrading small-chain AHLs can almost always degrade medium- and long-chain AHLs. A recent study, wherein 12 QQ bacteria were identified, showed that these bacteria could degrade a variety of different AHLs, but none of them could degrade C4-AHLs (Torres et al., 2016). This suggests that future studies in search of QQ bacteria should primarily focus on identifying bacteria capable of degrading small-acyl-chain AHLs.

Although QQ activity has been observed in either cell lysate or cell-free supernatant (Uroz et al., 2005; Shepherd and Lindow, 2009), to the best of our knowledge, it has not been detected in both fractions. The QQ activity found in both the cell lysate and supernatant of VG1 might represent a new class of QQ enzymes (Supplementary Figure 2B). However, it is possible that QQ molecules were released into the supernatant during sample preparation. Our results showing that the cell lysates of VG1 retain QQ activity even after heating at 105°C (data not shown) appear to contradict the heat killing of whole cells that can result in the loss of QQ activity. The exact reason for this observation is unknown, but it is possible that in case of cell lysates, the QQ enzyme can reform its 3D structure when cooled after heating. Fractionation of VG1 cell lysates with the 10-kDa-filter rule out the possibility that QQ is caused by small-molecular-weight metabolites that could be heat resistant. Unexpectedly, for VG12 and NV9, QQ activity was lost upon cell lysis. It is possible that the QQ enzymes of these strains are sensitive to our methods of cell disruption (sonication) or that these enzymes need certain factors/conditions for their activity, which are lost on cell lysis.

All QQ-positive isolates identified in this study belong to Proteobacteria (Supplementary Figure 4 and **Table 1**). These results are consistent with those of previous reports, where majority of the QQ bacteria identified were also Proteobacteria (Romero et al., 2011; Tan et al., 2015; Saurav et al., 2016; Torres et al., 2016). This is not surprising because Proteobacteria is predominant in various marine environments (Gonzalez and Moran, 1997).

Although disputed, it has been suggested that AHL-acylases are not active against small-acyl-chain AHLs such as C4-AHLs (Shepherd and Lindow, 2009; Czajkowski et al., 2011). If this is correct, then degradation of C4-AHLs and restoration of the degraded 3OXOC10-AHLs after acidification suggest that the QQ activity observed in NV9 is primarily caused by lactonase (**Figures 2A,B**). Genomic annotation of NV9 identified one ORFs (NV9_000000104) (Supplementary Table 2) that belongs to the MBL superfamily, which could be responsible for the observed QQ activity. In VG12, although the acidification of degraded AHLs restored only 2% of AHLs (**Figure 2B**), the ability of VG12 to effectively degrade C4-AHLs suggests lactonase activity (**Figure 2A**). It is possible that, in case of VG12, the hydrolyzed lactone ring of 3OXOC10-AHLs was further modified and was unable to reform the lactone ring. Furthermore, the prediction of only AHL-lactonases in the genome sequence of VG12 (**Table 2** and Supplementary Table 2) suggests that lactonases are responsible for QQ activity. Similarly, the genome sequence of a close relative of VG12, namely, *Labrenzia alba* CECT 755, carries only AHL-lactonases (CTQ52848.1, CTQ54016.1, CTQ52453.1, CTQ55013.1, and CTQ55918.1); no AHL-acylase was detected. For VG1, although both AHL-lactonases and acylases are predicted in the genome sequence (**Table 2** and Supplementary Table 2), its inability to degrade C4-AHLs (**Figure 2A**) and inability to relactonize 3OXOC10-AHLs after acidification (**Figure 2B**) suggest that AHL-acylases are responsible for QQ activity in this case. Interestingly, unlike VG1, the genome annotation of *Erythrobacter* species such as *E. longus* strain DSM 6997 (GenBank: JMIW0000000.1), *Erythrobacter* sp. HL-111 (GenBank: LT629743.1), and *E. citreus* strain LAMA915 (GenBank: JYNE00000000.1) show only AHL-acylases (KEO91396.1, SDS44800.1, SDT09981.1, and KNH01491.1) while no AHL-lactonase was detected in these bacteria. However, this difference might be caused by the different annotation methods/pipelines used. It is important to note that some recently discovered QQ enzymes did not show any sequence homology to the typical AHL-lactonases and acylases (Torres et al., 2017). Hence, it remains possible that the observed QQ activity is caused by a new class of enzymes.

The time-course experiment showed that VG1 can quickly degrade AHLs, closely followed by VG12 (**Figure 3**). We used 3OXOC10-AHLs for this assay, and it is possible that slow degradation by NV9 reflects its specificity for AHLs with a certain kind of acyl chains.

None of the QQ isolates was able to completely inhibit biofilm formation (**Figure 4A**), may be because biofilm formation is a complex process involving many factors (Flemming et al., 2016). We also tested QQ isolates for their ability to degrade the 3OXOC12-AHLs produced by PAO1 (**Figure 4B**), because 3OXOC12-AHLs lie higher in the hierarchy of the QS signaling cascade and regulate the expression of other QS molecules (C4-AHLs), production of virulence factors, and formation of biofilms (Whitehead et al., 2001; Williams and Camara, 2009). The observed ineffective degradation of 3OXOC12-AHLs and biofilm inhibition might be caused by certain PAO1 metabolites that inhibited the QQ activity of our isolates. It is also possible that 3OXOC12-AHLs are not a preferred substrate for our QQ isolates. A significant reduction in biofilm formation by VG12 might be the result of effective degradation of C4-AHLs caused by this isolate (**Figures 2A, 4A**). Based on our results, VG12 appears to be best isolate among others for the inhibition of biofilm formation and kinetics and diversity of AHL degradation (**Figures 1–3, 4A**). It also appears to be the best candidate for future studies employing bacteria as an anti-biofouling agent.

Quorum quenching alone might not completely abolish bacterial infections and biofilms, but it can be used in combination with other antimicrobial agents to achieve desired results. Combinatorial therapies are gaining importance because no single therapy or drug can effectively control bacteria for longer time periods, given that the bacteria will eventually develop resistance (Fischbach, 2011). Furthermore, QQ enzymes can confer resistance against antibiotics (Kusada et al., 2017). Therefore, improved understanding of these enzymes will provide opportunities to overcome such resistance.

In this study, we found bacteria belonging to three different genera, namely, *Erythrobacter, Labrenzia*, and *Bacterioplanes*, that can degrade AHLs. Although extracted metabolite-based QQ activity has been described for *Erythrobacter* and *Labrenzia* (Saurav et al., 2016), the bacteria identified in this study represent a new species whose QQ activity has not been described before. We have identified potential QQ genes and our future studies will focus on cloning these genes and investigating their mechanism of action.

## Author Contributions

ZR and TL designed the experiments and wrote and revised the manuscript. ZR performed the experiments.

## Conflict of Interest Statement

The authors declare that the research was conducted in the absence of any commercial or financial relationships that could be construed as a potential conflict of interest.

## Footnotes

1 https://bit.ly/2uoXhX1

2 https://bit.ly/2mr70HU

3 https://bit.ly/2NpfrPA
