# Supplementary material for: Quorum-Quenching Bacteria Isolated From Red Sea Sediments Reduce Biofilm Formation by Pseudomonas aeruginosa
Source: Front Microbiol. 2018 Jul 17;9:1354. doi: 10.3389/fmicb.2018.01354 (PMC6057113; doi:10.3389/fmicb.2018.01354)
Supplement: Supplementary file 1 [file Image_1.pdf]

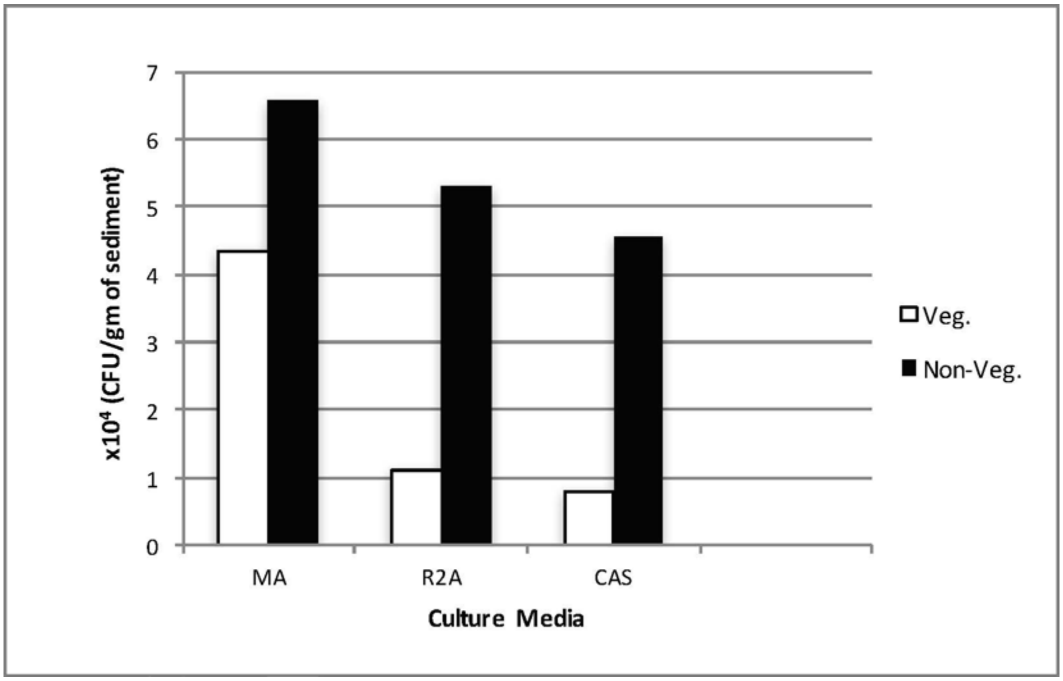

Supp. Figure 1. **Total CFUs per gram of sea sediment**

The total number of CFU obtained from 1 gram of sea sediment on different culture media are shown. White bars represent vegetative samples and black bars represent non-vegetative sea sediment samples. MA represents marine agar and CAS denotes Casamino acid agar.
