# Supplementary material for: Quorum-Quenching Bacteria Isolated From Red Sea Sediments Reduce Biofilm Formation by Pseudomonas aeruginosa
Source: Front Microbiol. 2018 Jul 17;9:1354. doi: 10.3389/fmicb.2018.01354 (PMC6057113; doi:10.3389/fmicb.2018.01354)
Supplement: Supplementary file 2 [file Image_2.PDF]

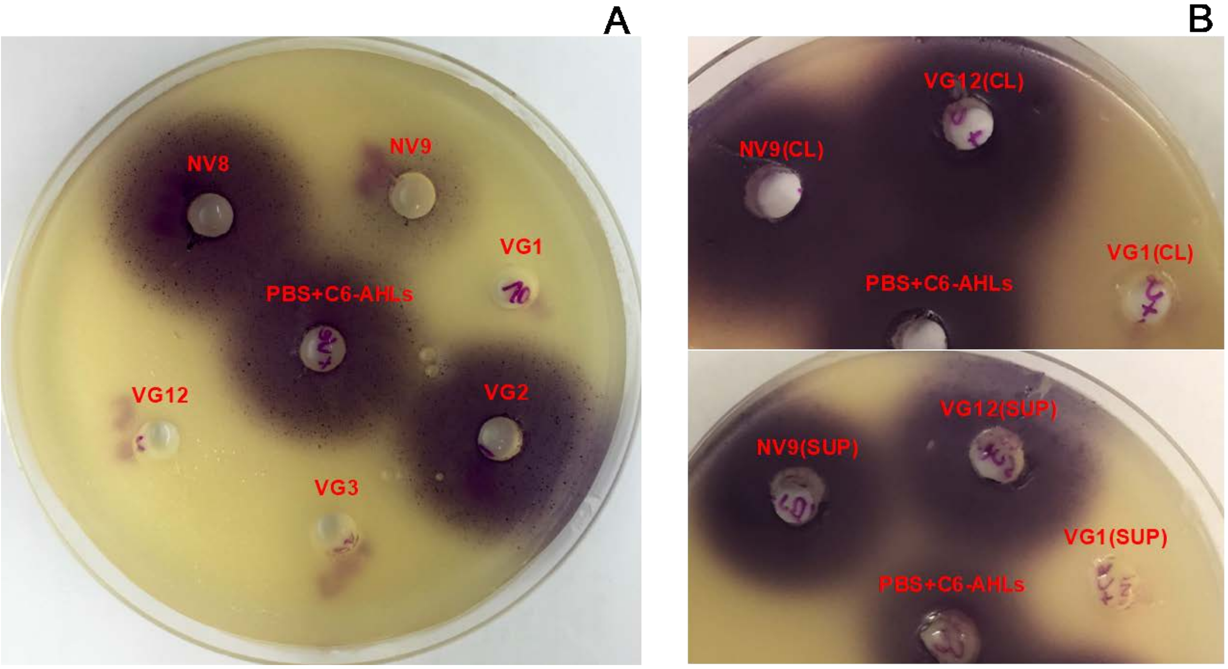

Supp. Figure 2. **Solid plate assay for the detection of QQ activity in marine bacteria**

(A) This is a representative image of QQ strains showing complete (VG1, VG3, and VG12) or partial (NV9) or no degradation (VG2 and NV8) of C6-AHLs. PBS with C6-AHLs was used as the positive control. **(B)** Localization of QQ activity, either in the cell lysates (top panel) or in the supernatant (bottom panel), is shown. QQ activity for VG1 was detected in both cell lysate and supernatant.
