# Supplementary material for: Quorum-Quenching Bacteria Isolated From Red Sea Sediments Reduce Biofilm Formation by Pseudomonas aeruginosa
Source: Front Microbiol. 2018 Jul 17;9:1354. doi: 10.3389/fmicb.2018.01354 (PMC6057113; doi:10.3389/fmicb.2018.01354)
Supplement: Supplementary file 3 [file Image_3.PDF]

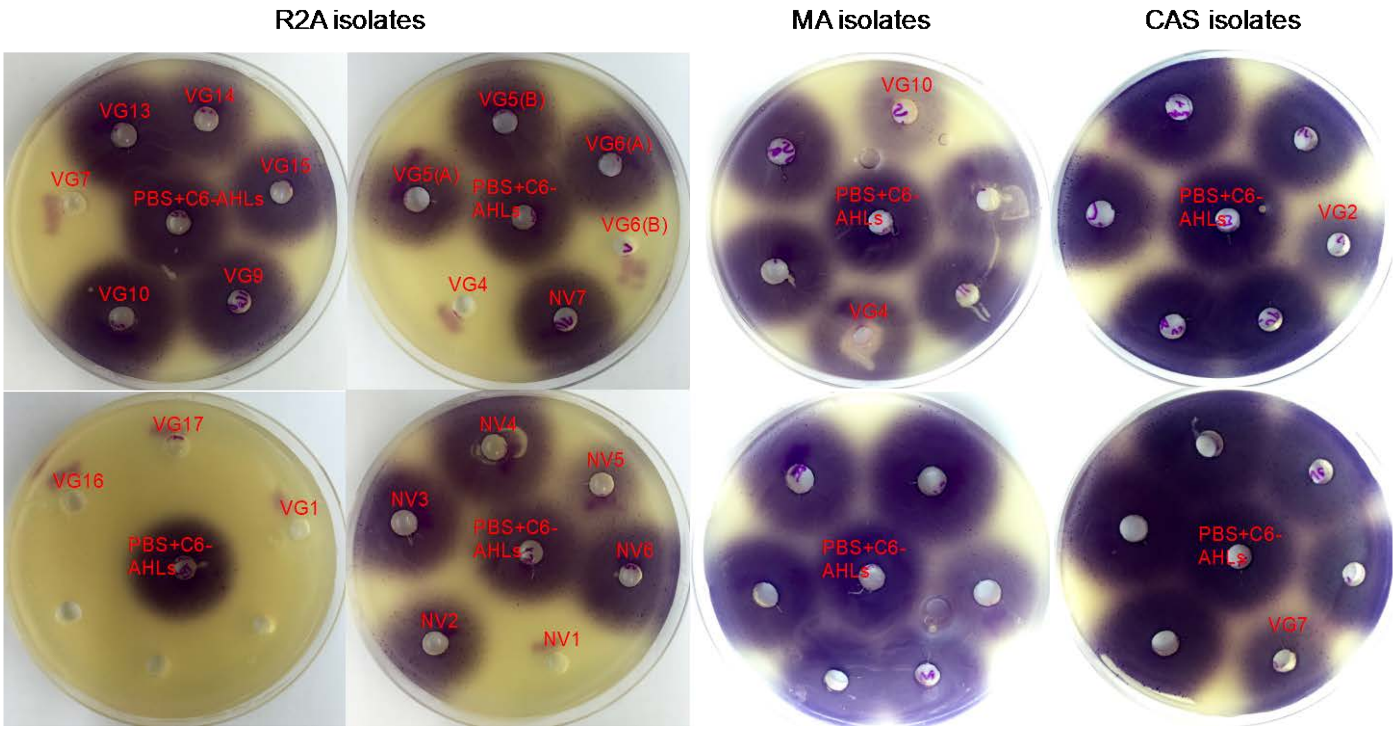

Supp. Figure 3. **Screening of bacterial isolates for QQ activity, based on the *C. violaceum*-based assay**

Presence of purple halos around the wells indicated bacterial isolates with no QQ activity, while the absence of halos indicated AHL degradation. PBS with C6-AHLs was used as negative control. MA, R2A, and CAS represent the different media used to isolate the test isolates. The assay was repeated thrice for positive strains. Representative images are shown.
