# Supplementary material for: Quorum-Quenching Bacteria Isolated From Red Sea Sediments Reduce Biofilm Formation by Pseudomonas aeruginosa
Source: Front Microbiol. 2018 Jul 17;9:1354. doi: 10.3389/fmicb.2018.01354 (PMC6057113; doi:10.3389/fmicb.2018.01354)
Supplement: Supplementary file 4 [file Image_4.PDF]

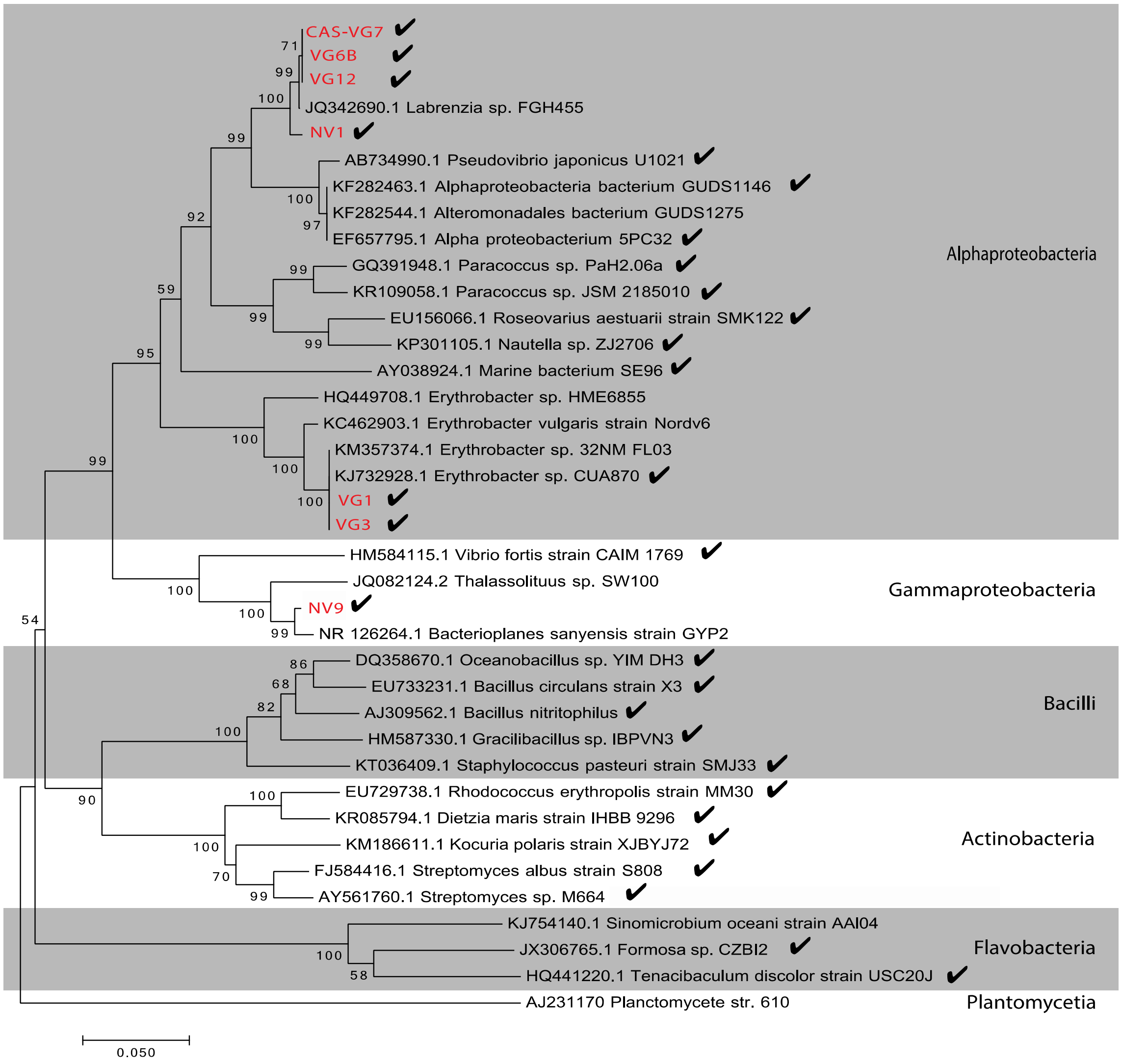

Supp. Figure 4. **Phylogenetic tree, based on the neighbor-joining method, of QQ-positive isolates identified in this study and those reported in literature**

The evolutionary relationship of 38 bacterial isolates was deduced based on 16S-rRNA gene sequences. All positions containing gaps and missing data were removed. A total of 619 positions were included in the final dataset. The bootstrap consensus tree was inferred from 500 replicates. Numbers at the nodes indicate the percentage level of bootstrap support; only values above 50% are shown. QQ-positive bacteria isolated in this study are shown in red. QQ-positive bacteria reported in literature are indicated by (✔).
