# Supplementary material for: Quorum-Quenching Bacteria Isolated From Red Sea Sediments Reduce Biofilm Formation by Pseudomonas aeruginosa
Source: Front Microbiol. 2018 Jul 17;9:1354. doi: 10.3389/fmicb.2018.01354 (PMC6057113; doi:10.3389/fmicb.2018.01354)
Supplement: Supplementary file 5 [file Table_1.DOCX]

Supp. Table 1: Identification of QQ positive strains based on 16S-rRNA gene sequencing.

| **Isolate** | **Closest match** | **Accession number** | **Phylogenetic branch** | **Seq. length** |
| --- | --- | --- | --- | --- |
| VG1 | *Erythrobacter flavus* SW-52 (100%) | AF500005.1 | Alphaproteobacteria | 1381 |
| VG3 | *Erythrobacter* sp. JL-378 (99%) | DQ285076.1 | Alphaproteobacteria | 1373 |
| VG6B | *Labrenzia alba* M2B125 (99%) | LN812985.1 | Alphaproteobacteria | 1373 |
| VG12 | *Alphaproteobacterium* JL001 (99%) | AY584527.1 | Alphaproteobacteria | 1377 |
| CAS-VG7 | *Alphaproteobacterium* JL001 (99%) | AY584527.2 | Alphaproteobacteria | 1378 |
| NV1 | *Labrenzia* sp. R-66638(99%) | KT185115.1 | Alphaproteobacteria | 802 |
| NV9 | *Bacterioplanes sanyensis* GYP-2 (99%) | NR_126264.1 | Gammaproteobacteria | 1431 |
