# Supplementary material for: Quorum-Quenching Bacteria Isolated From Red Sea Sediments Reduce Biofilm Formation by Pseudomonas aeruginosa
Source: Front Microbiol. 2018 Jul 17;9:1354. doi: 10.3389/fmicb.2018.01354 (PMC6057113; doi:10.3389/fmicb.2018.01354)
Supplement: Supplementary file 6 [file Table_2.DOCX]

Supp. Table 2: Genomic IDs of ORFs belonging to Metallo Beta Lactamase (MBL) and amidase family of proteins, predicted by either UniProt or KEGG databases.

| **Strains** | | **MBL** | **Amidase** |
| --- | --- | --- | --- |
| VG1 | VG1_000001716 | | VG1_000001506 |
|  | VG1_000003072 | |  |
| VG12 | VG12_000000644 | |  |
| NV9 | NV9_000000104 | | NV9_000004191 |
|  |  | | NV9_000002860 |
