# Supplementary material for: Quorum-Quenching Bacteria Isolated From Red Sea Sediments Reduce Biofilm Formation by Pseudomonas aeruginosa
Source: Front Microbiol. 2018 Jul 17;9:1354. doi: 10.3389/fmicb.2018.01354 (PMC6057113; doi:10.3389/fmicb.2018.01354)
Supplement: Supplementary file 7 [file Table_3.DOCX]

Supp. Table 3. Annotated genomic features and their counts are given.

| **Genome features** | **Counts** | | |
| --- | --- | --- | --- |
|  | **VG1** | **VG12** | **NV9** |
| ORFs | 3165 | 5993 | 4171 |
| rRNA | 6 | 9 | 12 |
| tRNA | 49 | 61 | 54 |
| RfamRNA | 38 | 48 | 32 |
| UniProt | 2913 | 5332 | 3649 |
| KEGG | 2218 | 4217 | 2884 |
| COG | 1942 | 3593 | 2295 |
| InterPro | 2291 | 4349 | 2992 |
| GO | 1612 | 2998 | 2111 |
| Total annotated genes | 3007 | 5452 | 3750 |
| Total Unassigned genes | 251 | 659 | 519 |
