# Supplementary material for: Quorum-Quenching Bacteria Isolated From Red Sea Sediments Reduce Biofilm Formation by Pseudomonas aeruginosa
Source: Front Microbiol. 2018 Jul 17;9:1354. doi: 10.3389/fmicb.2018.01354 (PMC6057113; doi:10.3389/fmicb.2018.01354)
Supplement: Supplementary file 8 [file Table_4.DOCX]

Supp. Table 4: Sequences of primers used for amplification of 16S-rRNA gene.

| Primer name | Sequence |
| --- | --- |
| 27F | 3′-AGAGTTTGATCMTGGCTCAG-5′ |
| 357F | 3′-CCTACGGGAGGCAGCAG-5′ |
| 785F | 3′-GGATTAGATACCCTGGTA-5′ |
| 785R | 3′-CTACCAGGGTATCTAATCC-5′ |
| 907R | 3′-CCGTCAATTCCTTTGAGTTT-5′ |
| 1492R | 3′-CTACGGCTACCTTGTTACGA-5′ |
